# Encapsulation state of messenger RNA inside lipid nanoparticles

**DOI:** 10.1016/j.bpj.2021.03.012

**Published:** 2021-03-25

**Authors:** Mark L. Brader, Sean J. Williams, Jessica M. Banks, Wong H. Hui, Z. Hong Zhou, Lin Jin

**Affiliations:** 1 Moderna, Inc., Cambridge, Massachusetts; 2 Department of Microbiology, Immunology, and Molecular Genetics, Los Angeles, Los Angeles, California; 3 California NanoSystems Institute, University of California, Los Angeles, Los Angeles, California

## Abstract

Understanding the structure of messenger RNA (mRNA) lipid nanoparticles, and specifically the microenvironment of the mRNA molecules within these entities, is fundamental to advancing their biomedical potential. Here, we show that a permeating cationic dye, thionine, can serve as a cryogenic electron microscopy contrasting agent by binding selectively to encapsulated mRNA without disturbing lipid nanoparticle morphology. Cryo-electron microscopy images identify the mRNA location, revealing that mRNA may exist within solvent-filled cavities or may be substantially lipid associated.

## Significance

The clinical utility of messenger RNA (mRNA) vaccines delivered in lipid nanoparticles has recently been highlighted by their use in SARS-CoV-2 vaccines from Pfizer-BioNTech (BNT162b2) and Moderna (mRNA-1273). The lipid nanoparticle is a complex entity in terms of structural and physical attributes that influence biological efficacy and pharmaceutics. One of the most fundamental yet elusive questions has been the nature and location of the mRNA payload inside the lipid nanoparticle. Elucidating this detail via direct experimental methods has been a major objective in the field of RNA delivery. Here, we show that a combination of dye-binding and cryo-electron microscopy pinpoints the mRNA location, providing new insights into its encapsulation state and chemical microenvironment.

## Introduction

There is considerable interest in using messenger RNA (mRNA) for vaccine and therapeutic applications (1, 2, 3, 4). A major challenge with this concept is achieving efficient delivery of mRNA into cells where it can affect expression of the desired protein or, in the case of vaccines, a disease-specific antigen protein. Lipid nanoparticles (LNPs) have emerged as the most promising nonviral delivery vehicle for exogenous mRNA (5,6). The LNP is a complex nanostructured entity that serves to protect the delicate RNA molecule from the harshly degrading nuclease environment in vivo while facilitating intracellular delivery. It comprises several lipid components, including an ionizable lipid that plays a central role in delivery efficacy (7, 8, 9). Entrapment of RNA is achieved by combining RNA with lipids at an acidic pH (10) at which the ionizable lipid is positively charged, thus ensuring a charge-driven interaction with the negatively charged nucleic acid. After a period of maturation, pH adjustment above the pKa of the ionizable lipid results in a near-neutral surface charge desirable for clinical administration (10). The incorporation of a pegylated lipid achieves a sterically stabilized core shell nanoparticle. Thus, the internal and external molecular architectures are both implicated in influencing biological activity and pharmaceutics. Although it is clear that the different lipids are not distributed uniformly throughout the LNP, details of the assembly and internal structure remain unsettled, especially in the case of mRNA, for which various models have been proposed and continue to be debated (11, 12, 13, 14, 15, 16). In the case of LNPs containing short interfering RNA (siRNA), small angle x-ray scattering and cryo-electron microscopy (cryo-EM) data have been interpreted as evidence of a multilamellar structure in which the siRNA molecule is sandwiched between bilayer lipid assemblies (10,17). However, the situation is less clear when LNPs containing the much larger mRNA molecule are also considered. Intriguingly, mRNA-LNPs are known to form both spherical and highly nonspherical morphologies (9,11,16,18,19), but the origins and practical implications of these morphological variants remain unclear. Molecular simulation studies (20) indicate that the LNP structure may involve solvent pockets, and experimental studies have shown nonspherical morphologies to be associated with segregation of the bilayer-forming 1,2-distearoyl-*sn*-glycero-3-phosphocholine (DSPC) component, resulting in pronounced blebs (11). Despite significant efforts to pinpoint the nucleic acid payload, the precise location of mRNA molecules within LNPs has not heretofore been unambiguously identified via direct experimental methods.

One difficulty with the analysis of LNPs is that many biophysical techniques produce a globally averaged signal or have limited selectivity for the RNA molecule within the solid particle. Although cryo-EM is a powerful tool for visualizing LNPs, the mass density contrast alone is not distinctive enough to unambiguously resolve RNA from lipidic components. To overcome these limitations, we explored the concept of using an RNA-binding dye to stain LNPs in a somewhat analogous manner to classical cell staining with membrane-permeable dyes (21).

## Results and Discussion

**Figure 1:**
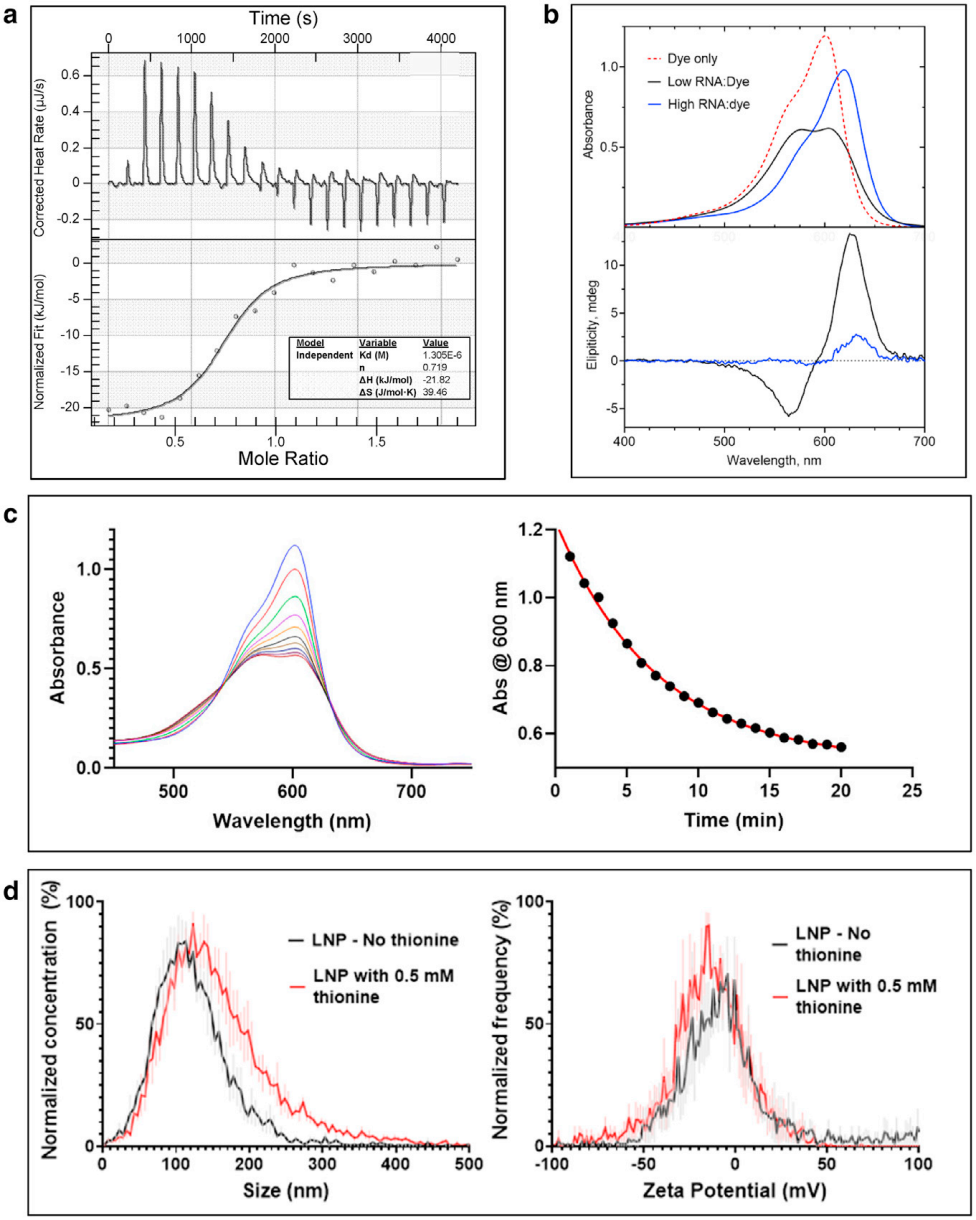
Characterization of dye-binding and permeation. (*a*) Isothermal titration calorimetry of thionine titrated into mRNA. (*Top*) Raw data. (*Bottom*) Integrated heats of each injection versus molar ratio of thionine/nucleotide together with a fit using a one-site binding model. (*b*) Optical signatures of the thionine-mRNA binding interaction. Visible absorption spectra (*top*) of thionine at 0.0255 mM recorded in the absence of mRNA and in the presence of mRNA at low (4) and high (160) nucleotide/dye molar ratios (P/D). Corresponding circular dichroism (CD) spectra (*bottom*) show that at low P/D, the thionine absorption bands are resolved into negative and positive CD bands with extrema at 565 and 628 nm, respectively, whereas at high P/D, the induced CD is weak and characterized by a single positive band at 632 nm. (*c*) Dye permeation kinetics corresponding to mRNA-LNP added to a thionine solution. (*Left*) Scanning kinetics showing the spectral change as a function of time with scans taken at 2-min intervals. (*Right*) Kinetic time course of 600 nm absorbance together with a fit (*red*) to a first-order process with a rate constant of k = 0.143 min^−1^. (*d*) Nanoparticle tracking analysis of mRNA-LNP in the presence and absence of 0.5 mM thionine, showing small or insignificant effects of thionine on particle size and charge. Data represent the mean and error of 3 independent samples. (*Left*) Size distribution. (*Right*) *ζ* potential distribution.

The binding of thionine, a cationic phenothiazinium dye, to an mRNA was characterized using isothermal titration calorimetry (Fig. 1 *a*). The binding isotherm exhibits a large exothermic enthalpy that may be approximated with a single-site model, yielding a stoichiometry of ∼0.7 dye molecules per nucleotide. The binding interaction produces distinctive changes in the thionine visible absorption spectrum that are dependent on the RNA/dye ratio (Fig. 1 *b*). This direct interaction of thionine with mRNA is confirmed by circular dichroism (CD) induced in the thionine absorption bands attributed to intimate contact of the achiral dye with the chiral mRNA molecule. Intercalation and electrostatic (outside) binding modes have been distinguished previously for phenothiazinium dyes binding to DNA (22) and RNA (23). The optical signatures of Fig. 1 *b* are consistent with the RNA/dye ratio-dependent signatures of these binding modes reported for transfer RNA (24). To characterize dye permeation into the LNP, we added LNP to an excess of thionine solution (Fig. 1 *c*). Scanning kinetics show spectral changes indicative of electrostatic binding (Fig. 1 *c*), with two isosbestic points as direct evidence of a two-state system comprising free and bound dye at any wavelength. The spectral change follows first-order kinetics, which we interpret as indicative of thionine permeation into the LNP via passive diffusion. A nanoparticle tracking analysis (NTA) of LNPs in the presence and absence of thionine indicates highly comparable particle size and *ζ* potential distributions (Fig. 1 *d*). We infer from the *ζ* potential result that thionine binding to the mRNA within the LNP does not perturb surface charge and likely occurs via a counterion exchange process.

**Figure 2:**
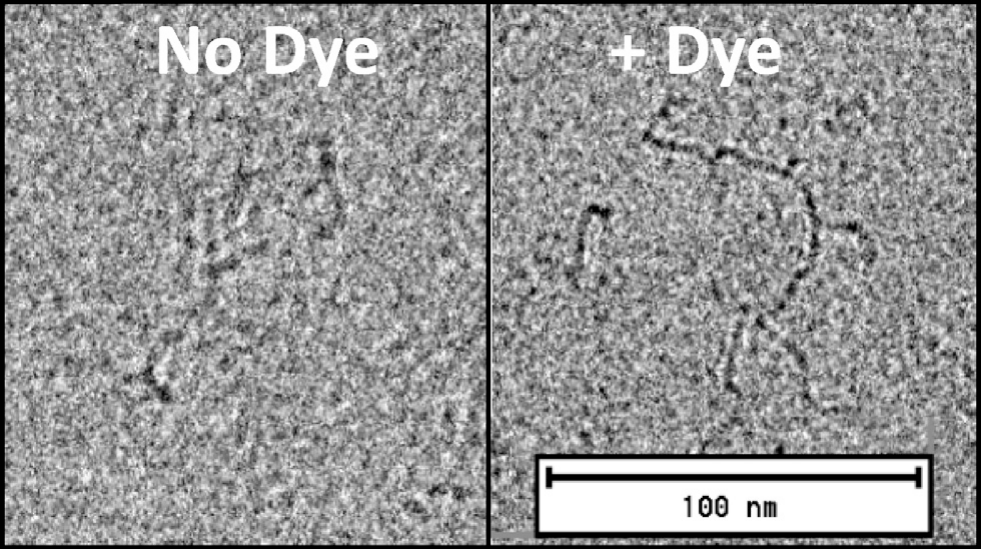
Visualizing free mRNA. Images show contrast enhancement using thionine of a 4000 nucleotide mRNA molecule. In the absence of dye (*left*), the resolution is comparable to published examples (25), whereas in the presence of 0.1 mM thionine (*right*), extended strand and molecular branching is more clearly discernible.

**Figure 3:**
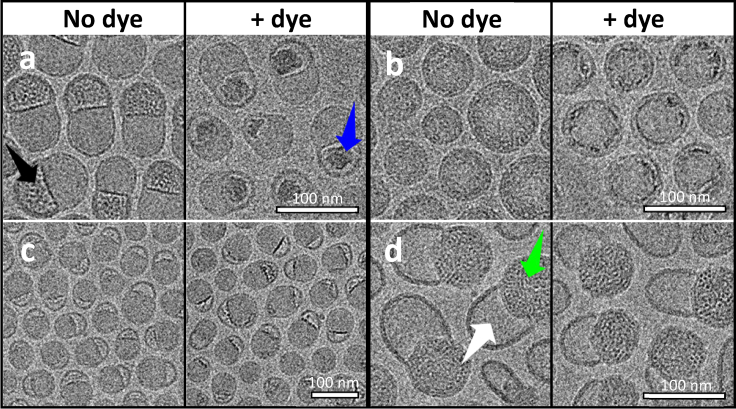
Locating encapsulated mRNA in LNPs by cryo-EM. The effect of thionine on mRNA-LNPs of different morphologies reveals that lipid-dissociated mRNA may reside in bleb compartments (*a*) or may be more lipid-associated in spherical (*b*) or less prominently blebbed particles (*c*). The black arrow in (*a*) indicates the distinctive mottled mass density of mRNA inside the bleb cavity, which itself is distinguished by a thick, dark periphery. The blue arrow indicates the significant contrast enhancement that occurs when thionine dye is present, thereby identifying mRNA within the bleb. (*d*) Charge-driven migration of mRNA from the bleb into the body of the LNP. When the mRNA-LNP sample of (*a*) (no dye) was dialyzed into pH 5 buffer, the images of (*d*) resulted. The mottled density is now associated with the body of the LNP (*green arrow*), leaving the bleb cavity devoid of mRNA (*white arrow*). Addition of thionine to this sample produced no notable contrast change (*right*), indicating that thionine did not displace lipid.

The binding specificity of thionine for mRNA, together with its ability to permeate the LNP, raised the possibility of exploiting these properties for cryo-EM contrast enhancement. A cryo-EM image of mRNA with thionine shows a branched character consistent with the secondary structural nature of large single-stranded RNA molecules (25), whereas poorly resolved images were obtained under the same imaging conditions in the absence of thionine (Fig. 2). To examine the LNP structure, we selected a prototypical set of morphological variants for characterization in the presence and absence of thionine (Fig. 3, *a*–*c*). These images show that thionine enhanced the contrast of a specific region of the LNP, whereas the rest of the particle remained unchanged. Taken together with the calorimetric and spectroscopic data herein, these images provide strong evidence of an RNA-specific contrast enhancement effect of thionine. As a control, cryo-EM images of LNPs prepared in the absence of mRNA showed no enhancement when stained with thionine (Data S1). Furthermore, it is apparent from the dye-stained image of the nonspherical LNP specimen (Fig. 3 *a*) that mRNA resides inside the bleb compartment and that the distinctive mottled mass density apparent in the nondye image can now be assigned to mRNA. This interpretation differs from that of Leung and co-workers (11), who reported a strikingly similar cryo-EM image yet assigned the mRNA location to the body of the particle adjoined to the solvent-filled bleb. To gain further evidence that this distinctive mottled cryo-EM motif may be assigned to mRNA, the LNP of Fig. 3 *a* was dialyzed overnight into pH 5 buffer, causing a major change to its overall morphology (Fig. 3 *d*). Now recognizing that the mottled mass density inside the bleb corresponds to mRNA, it is apparent that the pH 5 condition caused the mRNA to reassociate with the lipidic body of the LNP, leaving the bleb compartment empty. This observation aligns with the widely accepted understanding of a charge-driven basis for LNP assembly at low pH—evidently, adjustment to pH 5 has caused the amino lipid to become positively charged, thereby driving reassociation with the negatively charged mRNA. Addition of thionine to the pH 5 sample caused no discernable contrast enhancement (Fig. 3 *d*), indicating that mRNA is complexed with charged lipid at this pH and thionine does not displace it. It is now apparent that mRNA may exist fully encapsulated within the spherical particle (Fig. 3 *b*) or dissociated within a large bleb in the highly nonspherical particle (Fig. 3 *a*) or, alternatively, may be in an intermediate state (Fig. 3 *c*). The cryo-EM images also indicate that blebs may be empty or mRNA loaded, but without the benefit of mRNA-specific contrast enhancement, it has not previously been possible to recognize these distinctions. Although blebs have been widely referred to in the literature as structural defects, it is apparent that varying degrees of particle nonsphericity can also be viewed in the context of a continuum of states involving differing degrees of mRNA-lipid association. It has been well established that LNPs with a diversity of morphological features can be generated through both manipulation of rapid precipitation conditions and formulation composition (10,15,26). LNP assembly models described in the literature reflect the large number of degrees of freedom and complex interplay within this system. These structural models are based on unilamellar, bilamellar, multilamellar, and polymorphic or faceted lipid arrangements combined in various ways with nanostructured and homogeneous cores (9, 10, 11,13,15,20). Pinpointing the mRNA within these structures should help provide further insights into the applicability of these models.

**Figure 4:**
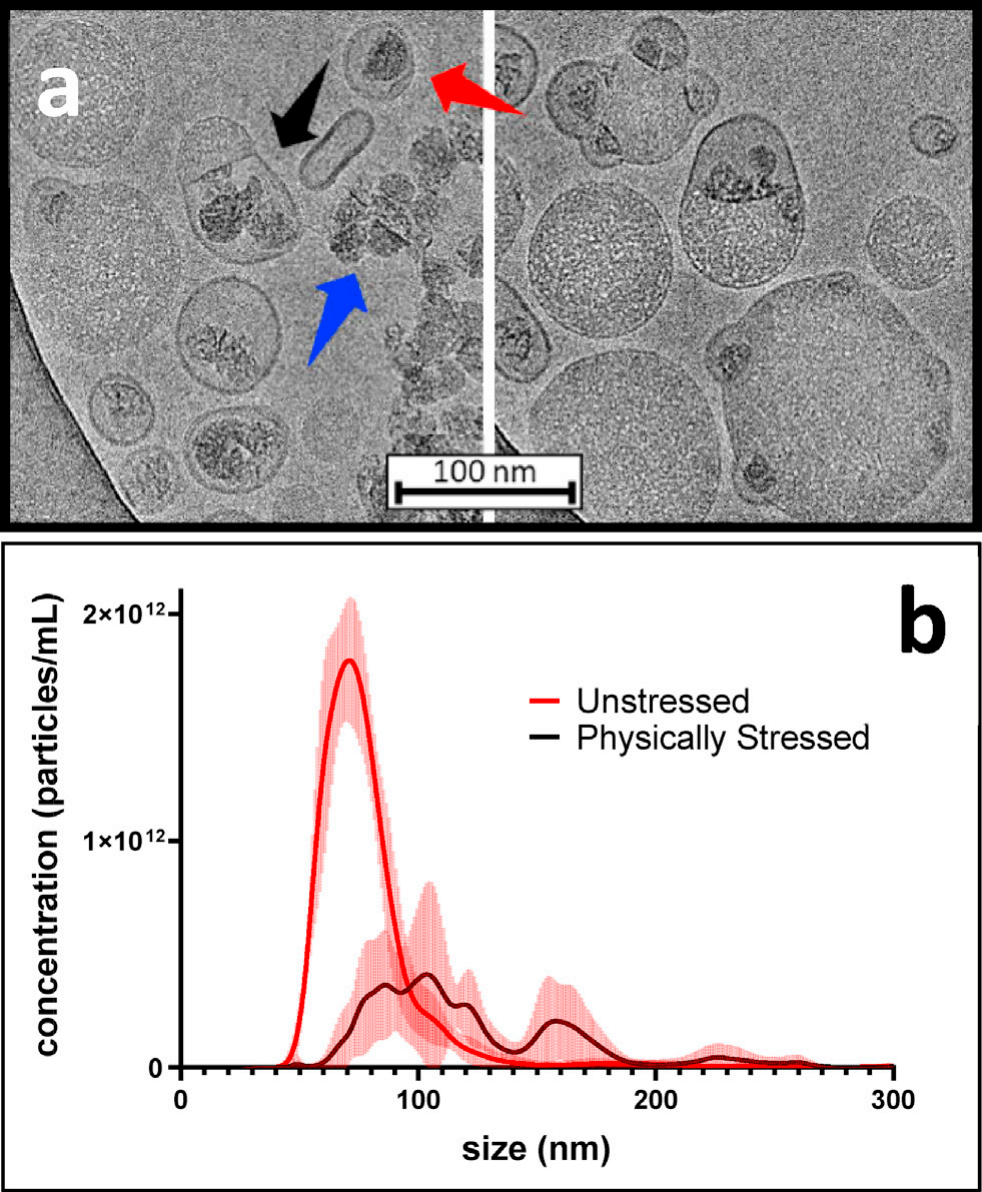
LNP physical degradation pathways. (*a*) Effects of physical stress on the LNP highlighted by dye. A dye-stained mRNA-LNP sample was subjected to multiple freeze-thaws. The resulting effects of this physical stress are evident, revealing aggregation (*right*), liberated mRNA (*blue arrow*), and the formation of liposomal structures containing mRNA (*red arrow*). The black arrow indicates an LNP that appears ready to burst. This image appears to capture a snapshot of how physical degradation leads to loss of mRNA encapsulation. (*b*) NTA size distribution profiles corresponding to the stressed and unstressed sample. Mean values with error bars are plotted. A cryo-EM image of the original unstressed LNP sample is provided as Data S2.

We also examined a physically stressed LNP using the dye-staining method (Fig. 4 *a*). These images show that bleb dissociation can occur, resulting in a liposomal structure with dye-bound mRNA encapsulated within. The dissociated bodies of the LNP are circular and exhibit a distinctive cryo-EM appearance, presumably due to depletion of the mRNA and DSPC components. It is also apparent that physically stressed LNPs can associate to form larger submicron particles and can undergo vesicle rupture. These images thus provide a nanoscale snapshot of physical degradation pathways of LNPs, identifying aggregation and loss of encapsulation, which are phenomena relevant to mRNA-LNP pharmaceutical development.

## Conclusion

In summary, we report that thionine staining in conjunction with cryo-EM selectively pinpoints lipid-dissociated mRNA within LNPs, representing a “nanoscale Rosetta Stone” that reveals new insights into LNP structure. Recognizing that mRNA can dissociate from the charged lipid to reside within a solvent-filled bleb compartment has direct implications for the chemical microenvironment of the mRNA and confers a new significance to this liposomal-like LNP structural domain.

## Author contributions

M.L.B. conceived and supervised the project. S.J.W., J.M.B., L.J., and M.L.B. prepared samples and collected and analyzed spectroscopic and calorimetric data. W.H.H. and Z.H.Z. collected cryo-EM data. M.L.B. wrote the manuscript, and all authors read, revised, and approved the manuscript.

## Supporting material

Document S1. Supporting materials and methods and Data S1 and S2

Document S2. Article plus supporting material

## References

[1] Kowalski P.S., Rudra A., Anderson D.G. (2019). Delivering the messenger: advances in technologies for therapeutic mRNA delivery. Mol. Ther.

[2] Karikó K. (2019). In vitro-transcribed mRNA therapeutics: out of the shadows and into the spotlight. Mol. Ther.

[3] Sahin U., Karikó K., Türeci Ö. (2014). mRNA-based therapeutics--developing a new class of drugs. Nat. Rev. Drug Discov.

[4] Jackson L.A., Anderson E.J., Beigel J.H., mRNA-1273 Study Group (2020). An mRNA vaccine against SARS-CoV-2 - preliminary report. N. Engl. J. Med.

[5] Guan S., Rosenecker J. (2017). Nanotechnologies in delivery of mRNA therapeutics using nonviral vector-based delivery systems. Gene Ther.

[6] Reichmuth A.M., Oberli M.A., Blankschtein D. (2016). mRNA vaccine delivery using lipid nanoparticles. Ther. Deliv.

[7] Semple S.C., Akinc A., Hope M.J. (2010). Rational design of cationic lipids for siRNA delivery. Nat. Biotechnol.

[8] Whitehead K.A., Dorkin J.R., Anderson D.G. (2014). Degradable lipid nanoparticles with predictable in vivo siRNA delivery activity. Nat. Commun.

[9] Miao L., Li L., Anderson D.G. (2019). Delivery of mRNA vaccines with heterocyclic lipids increases anti-tumor efficacy by STING-mediated immune cell activation. Nat. Biotechnol.

[10] Gindy M.E., DiFelice K., Boardman D. (2014). Mechanism of macromolecular structure evolution in self-assembled lipid nanoparticles for siRNA delivery. Langmuir.

[11] Leung A.K., Tam Y.Y., Cullis P.R. (2015). Microfluidic mixing: a general method for encapsulating macromolecules in lipid nanoparticle systems. J. Phys. Chem. B.

[12] Yanez Arteta M., Kjellman T., Lindfors L. (2018). Successful reprogramming of cellular protein production through mRNA delivered by functionalized lipid nanoparticles. Proc. Natl. Acad. Sci. USA.

[13] Viger-Gravel J., Schantz A., Emsley L. (2018). Structure of lipid nanoparticles containing siRNA or mRNA by dynamic nuclear polarization-enhanced NMR spectroscopy. J. Phys. Chem. B.

[14] Oberli M.A., Reichmuth A.M., Blankschtein D. (2017). Lipid nanoparticle assisted mRNA delivery for potent cancer immunotherapy. Nano Lett.

[15] Eygeris Y., Patel S., Sahay G. (2020). Deconvoluting lipid nanoparticle structure for messenger RNA delivery. Nano Lett.

[16] Kulkarni J.A., Witzigmann D., Cullis P.R. (2019). Fusion-dependent formation of lipid nanoparticles containing macromolecular payloads. Nanoscale.

[17] Kulkarni J.A., Darjuan M.M., Cullis P.R. (2018). On the formation and morphology of lipid nanoparticles containing ionizable cationic lipids and siRNA. ACS Nano.

[18] Patel S., Ryals R.C., Sahay G. (2019). Lipid nanoparticles for delivery of messenger RNA to the back of the eye. J. Control. Release.

[19] Richner J.M., Himansu S., Diamond M.S. (2017). Modified mRNA vaccines protect against Zika virus infection. Cell.

[20] Rozmanov D., Baoukina S., Tieleman D.P. (2014). Density based visualization for molecular simulation. Faraday Discuss.

[21] Scarff C.A., Fuller M.J.G., Iadanza M.G. (2018). Variations on negative stain electron microscopy methods: tools for tackling challenging systems. J. Vis. Exp.

[22] Paul P., Kumar G.S. (2010). Toxic interaction of thionine to deoxyribonucleic acids: elucidation of the sequence specificity of binding with polynucleotides. J. Hazard. Mater.

[23] Khan A.Y., Suresh Kumar G. (2016). Spectroscopic studies on the binding interaction of phenothiazinium dyes, azure A and azure B to double stranded RNA polynucleotides. Spectrochim. Acta A Mol. Biomol. Spectrosc.

[24] Antony T., Atreyi M., Rao M.V.R. (1995). Interaction of methylene blue with transfer RNA--a spectroscopic study. Chem. Biol. Interact.

[25] Gopal A., Zhou Z.H., Gelbart W.M. (2012). Visualizing large RNA molecules in solution. RNA.

[26] Maeki M., Fujishima Y., Tokeshi M. (2017). Understanding the formation mechanism of lipid nanoparticles in microfluidic devices with chaotic micromixers. PLoS One.

