# Supplementary material for: Encapsulation state of messenger RNA inside lipid nanoparticles
Source: Biophys J. 2021 Mar 25;120(14):2766–70. doi: 10.1016/j.bpj.2021.03.012 (PMC8390897; doi:10.1016/j.bpj.2021.03.012)
Supplement: Document S1. Supporting materials and methods and Data S1 and S2 [file mmc1.pdf]

Biophysical Journal, Volume *120*

Supplemental information

Encapsulation state of messenger RNA inside lipid nanoparticles

Mark L. Brader, Sean J. Williams, Jessica M. Banks, Wong H. Hui, Z. Hong Zhou, and Lin Jin

SUPPLEMENTAL INFORMATION

MATERIALS AND METHODS

mRNA and LNP Samples. mRNA was synthesized in vitro by T7 RNA polymerase-mediated transcription from a linearized DNA template, which incorporates the 5′ and 3′ untranslated regions and a polyadenosine tail, as described previously(1, 2). LNPs were prepared with novel amino lipids and a modified ethanol-drop nanoprecipitation process, as described in Hassett et al(3). The spectroscopic and calorimetric data reported in Figure 1 were collected on mRNA and mRNA-LNP described in An et al(4). The images of Figure 3 correspond to LNPs prepared with a single(4) (Figure 3b,c and Figure 4) or dual(5) mRNA (Figures 3a,d).

Cryo-EM. To prepare cryo-EM grids, 2.5 μL of sample was applied to a Quantifoil 200 mesh grid, manually blotted for ~3-4 seconds with filter paper, and then plunged into liquid ethane. The image was collected on a FEI Tecnai TF20 high resolution Transmission Electron Microscope at an accelerating voltage of 200 kV using TVIPS EM-Menu program. The instrument is equipped with a 16-megapixel CCD camera. The nominal magnification used was 29,000 and 50,000 with 2 binning. LNP samples for cryo-EM were prepared at 0.5 mg/mL (mRNA content) in 20 mM Tris, pH 7.4 incorporating either 8% sucrose or 7% propylene glycol (w/w) and stored refrigerated prior to cryo-EM analysis. Thionine-stained LNP samples incorporated 0.5 mM thionine acetate high purity biological stain (Acros Organics). The mRNA sample of Figure 2 was prepared at 0.5 mg/mL. Thionine acetate (0.1 mM) was added immediately prior to flash-freezing for cryo-EM.

Isothermal Titration Calorimetry. Titration experiments were performed using a TA Instruments Affinity ITC with a Gold/Hastelloy reaction vessel. The reference cell was filled with water. Each titration experiment consisted of twenty, 2 μL injections of dye into LNP at 200-second intervals with a stirring speed of 350 rpm. A 300-second baseline was collected before the first injection. LNP and thionine solutions were prepared in 20 mM Tris, pH 7.4. The instrument software, NanoAnalyze version 3.7.0, was used for data analysis.

Optical Spectroscopy. Visible absorption spectra were collected using an Agilent Cary 100 UV-vis spectrophotometer and circular dichroism spectra were collected using a Jasco J-1500 circular dichroism spectrophotometer. Spectra were recorded in 10 mM Tris, 4% w/w sucrose, pH 7.4 at 20°C using 1 cm quartz cuvettes. Thionine concentration was determined using ε_{580}=54,800 $M^{-1}cm^{-1}$(6). Visible absorption spectra (400-800 nm) of thionine solution with added mRNA-LNP were collected using scanning kinetics mode with a 2-minute interval between scans. The thionine permeation kinetics curve was extracted by plotting the 600 nm absorbance value versus time, then analyzing with GraphPad Prism software (version 8.1.0) single phase exponential decay function.

Particle Size and Zeta Potential. Samples were analyzed for size and zeta potential (Figure 1d) using a ZetaView Nanoparticle Tracking Analyzer PMX-120 (Particle Metrix GmbH) or size only (Figure 4b) using a NanoSight NS300 (Malvern Panalytical Ltd, UK). Samples were diluted with 20 mM Tris, 8% sucrose, pH 7.4 to an mRNA concentration of $3.0\text{-}6.5 \times 10^{-6}$ mg/mL. Measurements were collected and analyzed using ZetaView version 8.05.12 SP1 software. For size distribution measurement, 11 positions were measured for 5 cycles in scatter mode using a 520 nm laser. For zeta potential, 5 cycles in scatter mode using a 520 nm laser were measured in continuous mode in the two stationary layer positions. The Smoluchowski approximation was used to calculate the zeta potential. The data presented in Figure 1d represent the mean and error of 3 independent samples.

REFERENCES

1. Richner, J. M., S. Himansu, K. A. Dowd, S. L. Butler, V. Salazar, J. M. Fox, J. G. Julander, W. W. Tang, S. Shresta, T. C. Pierson, G. Ciaramella, and M. S. Diamond. 2017. Modified mRNA Vaccines Protect against Zika Virus Infection. *Cell* 168:1114-1125.

2. Nelson, J., E. W. Sorensen, S. Mintri, A. E. Rabideau, W. Zheng, G. Besin, N. Khatwani, S. V. Su, E. J. Miracco, W. J. Issa, S. Hoge, M. G. Stanton, and J. L. Joyal. 2020. Impact of mRNA chemistry and manufacturing process on innate immune activation. *Science Advances* 6:eaaz6893.

3. Hassett, K. J., K. E. Benenato, E. Jacquinet, A. Lee, A. Woods, O. Yuzhakov, S. Himansu, J. Deterling, B. M. Geilich, T. Ketova, C. Mihai, A. Lynn, I. McFadyen, M. J. Moore, J. J. Senn, M. G. Stanton, Ö. Almarsson, G. Ciaramella, and L. A. Brito. 2019. Optimization of Lipid Nanoparticles for Intramuscular Administration of mRNA Vaccines. *Mol Ther Nucleic Acids* 15:1-11.

4. An, D., A. Frassetto, E. Jacquinet, M. Eybye, J. Milano, C. DeAntonis, V. Nguyen, R. Laureano, J. Milton, S. Sabnis, C. M. Lukacs, and L. T. Guey. 2019. Long-term efficacy and safety of mRNA therapy in two murine models of methylmalonic acidemia. *EBioMedicine* 45:519-528.

5. Jiang, L., J.-S. Park, L. Yin, R. Laureano, E. Jacquinet, J. Yang, S. Liang, A. Frassetto, J. Zhuo, X. Yan, X. Zhu, S. Fortucci, K. Hoar, C. Mihai, C. Tunkey, V. Presnyak, K. E. Benenato, C. M. Lukacs, P. G. V. Martini, and L. T. Guey. 2020. Dual mRNA therapy restores metabolic function in long-term studies in mice with propionic acidemia. *Nature Communications* 11:5339.

6. Köhler, G., S. Solar, and N. Getoff. 1980. Thionine Fluorescence Quenching by Metal Cations. *Zeitschrift für Naturforschung A* 35:1201.

SUPPLEMENTARY DATA

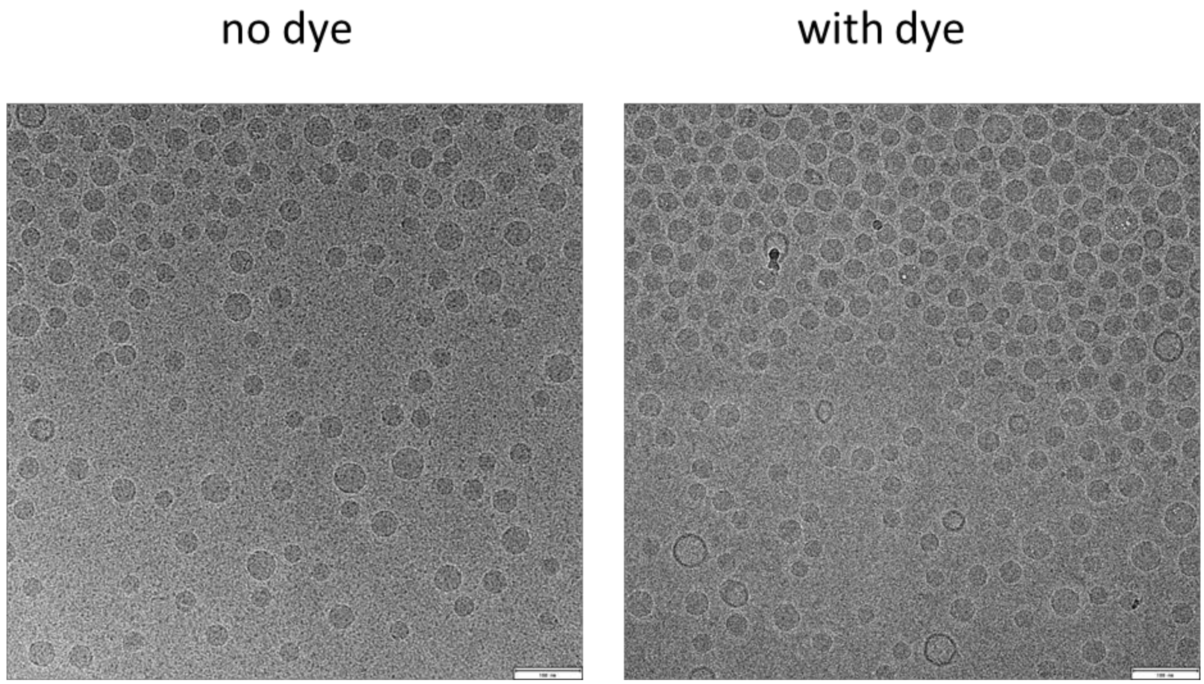

Supplementary Data S1. Images of LNP prepared without mRNA imaged in the absence of dye (Left) versus the presence of dye (Right). There is no discernable effect of dye on contrast within the particles supporting the interpretation that it is thionine-binding to the mRNA component of the mRNA-LNP that results in the localized darkening effect (scale bar, 100 nm).

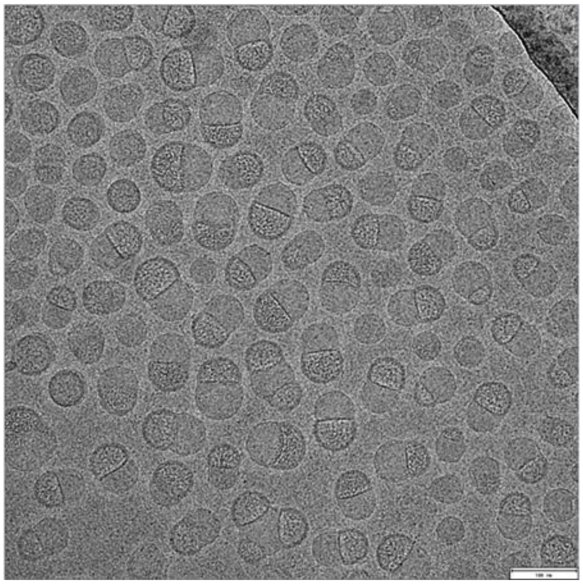

Supplementary Data S2. Image of the LNP sample studied in Figure 4 recorded prior to stress (scale bar, 100 nm).

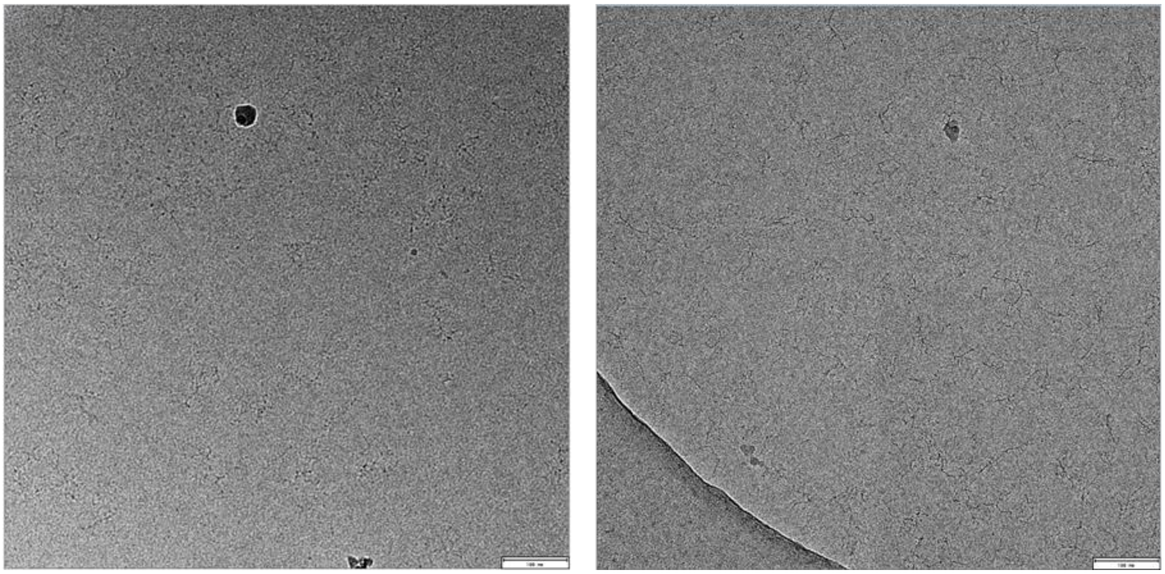

Uncropped images corresponding to Figure 2. No dye (Left) + dye (Right). (Scale bar, 100 nm)

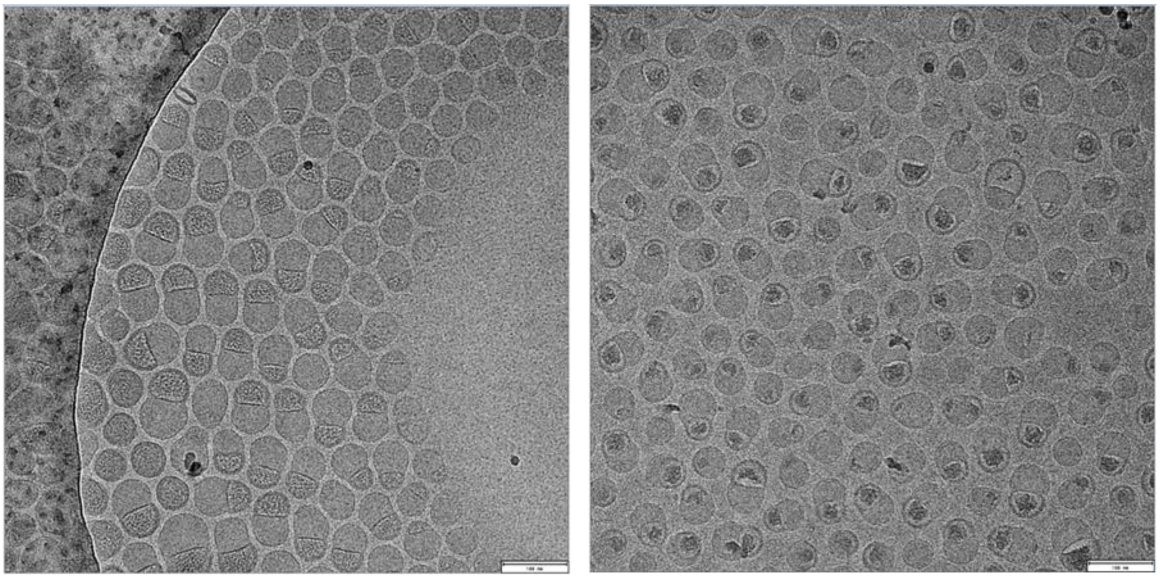

Uncropped images corresponding to Figure 3a. No dye (Left) + dye (Right). (Scale bar, 100 nm)

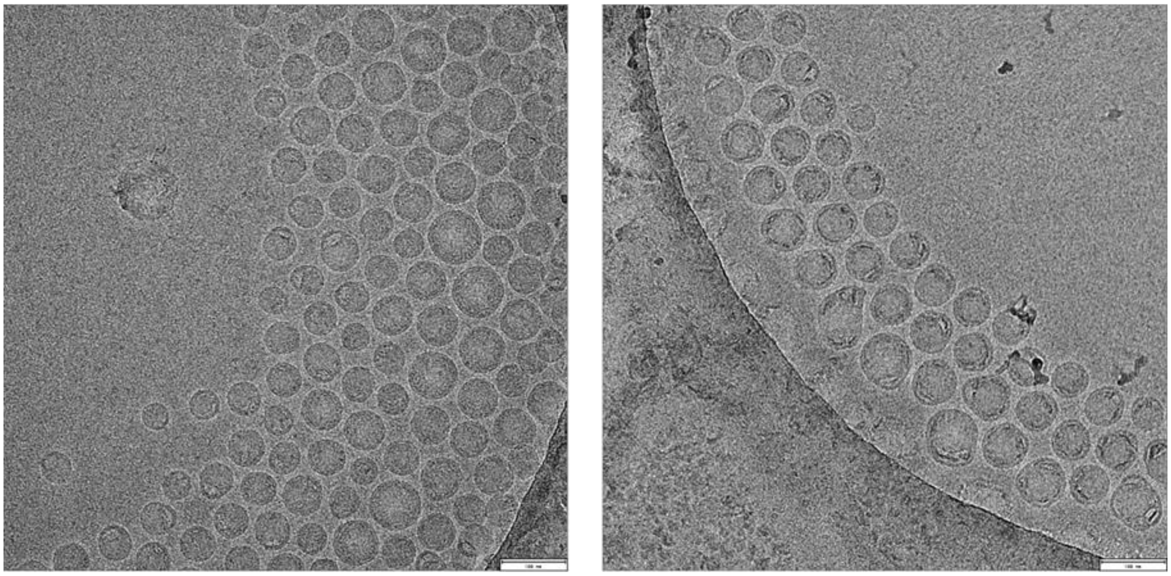

Uncropped images corresponding to Figure 3b. No dye (Left) + dye (Right). (Scale bar, 100 nm)

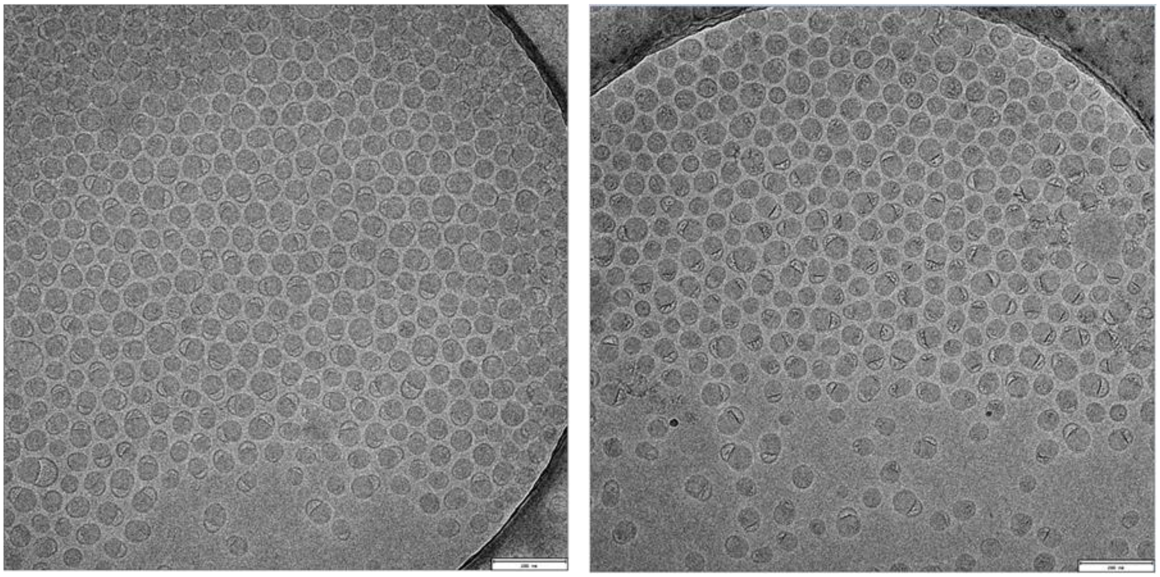

Uncropped images corresponding to Figure 3c. No dye (Left) + dye (Right). (Scale bar, 200 nm)

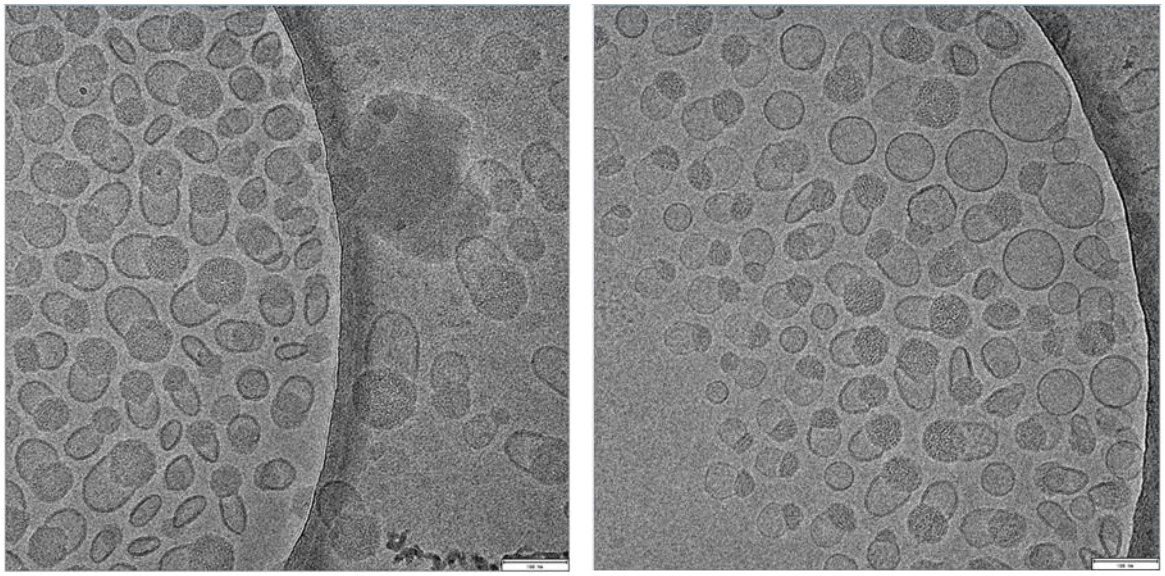

Uncropped images corresponding to Figure 3d. No dye (Left) + dye (Right). (Scale bar, 100 nm)

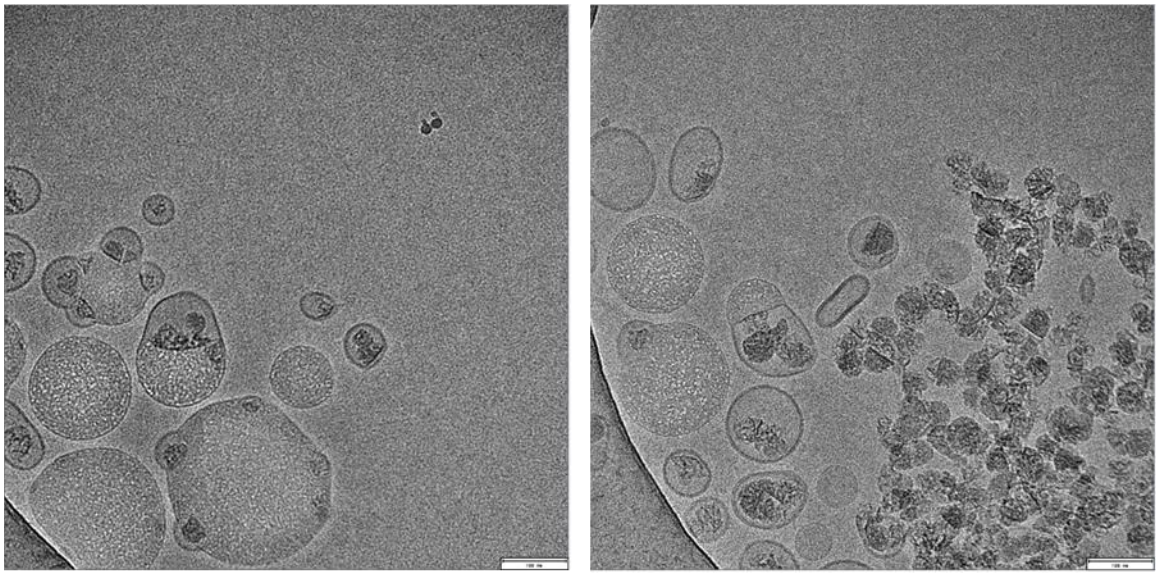

Uncropped images corresponding to Figure 4a. Both images + dye. (Scale bar, 100 nm)
